# A comparison of the quantitative evaluation of *in situ* parathyroid gland perfusion by indocyanine green fluorescence angiography and by visual examination in thyroid surgery

**DOI:** 10.20945/2359-3997000000219

**Published:** 2020-03-30

**Authors:** Erkan Yavuz, Aytac Biricik, Onur Olgac Karagulle, Candas Ercetin, Sinan Arici, Hakan Yigitbas, Serhat Meric, Ali Solmaz, Atilla Celik, Osman Bilgin Gulcicek

**Affiliations:** 1 Istanbul Bagcilar Training and Research Hospital Department of General Surgery Istanbul Turkey Istanbul Bagcilar Training and Research Hospital , Department of General Surgery , Istanbul , Turkey

**Keywords:** Thyroidectomy, parathyroid, hypoparathyroidism, SPY, Indocyanine green

## Abstract

**Objective:**

The most vital complications of thyroidectomy are recurrent nerve damage and hypocalcaemia. We aimed to compare the tissue perfusion scores (PS) of IG fluorescence angiography (IGFA) and visual examination by the surgeon after total thyroidectomy.

**Subjects and methods:**

Forty-three patients were accepted into the study. Localisation of the parathyroid gland (PG) was determined by the naked eye and scored in terms of tissue perfusion. The averages of fluorescent light intensities for each IGFA were calculated, the perfusions were scored and compared with the PS given by the surgeon. Biochemical parameters were noted.

**Results:**

37.2% of patients had autotransplanted PGs, according to their visual scores. The means of IGFA-PS for PGs scored as 0, 1 or 2 on visual inspection were 48.58 ± 4.49 [30-70], 89.65 ± 8.93 [36-144] and 158.76 ± 8.93 [70-253], respectively, which correlated with the visual PSs in a statistically significant manner (P < 0.0001). The predictive cut-off value for IGFA-PS was determined to be 70, given a visual PS of 0 (95% CI [0.72-0.85]), and this was interpreted to be a candidate cut-off point for the autotransplantation of PGs.

**Conclusion:**

IGFA scoring may be considered as an operative predictor, providing objective criteria to evaluate the tissue and blood perfusion of PGs after thyroidectomy. IGFA scoring may be considered to have value in minimising postoperative permanent hypoparathyroidism in patients.

## INTRODUCTION

The most vital complications of thyroidectomy are recurrent laryngeal nerve (RLN) damage and postoperative hypocalcaemia, and the most common cause of hypocalcaemia is post-surgery hypoparathyroidism (1,2). Hypocalcaemia may persist for more than 6 months after surgery; in which case it is termed permanent hypocalcaemia (3). Although the rate of permanent hypoparathyroidism has been reported to be between 0% and 43% in the literature (4,5), the current acceptable rate in endocrine surgery has been reported to be 1-2% (6). The best way to avoid the complication of hypoparathyroidism is careful and meticulous dissection, and ligation of the inferior thyroid artery branches separately at the thyroid capsule in order to prevent damage to parathyroid gland (PG) vascularisation. However, despite all precautions, unintentional parathyroidectomy during thyroidectomy can be detected at a rate of 9-19% (7). In the case of unintentional excision of PGs or impairment of the vascular supply of tissue, the gland’s functions may be preserved by parathyroid auto-transplantation (PA), but it may not be sufficient to decide which of the glands should be autotransplanted or left *in situ* (8).

Laser Doppler flowmetry was previously used for assessing parathyroid perfusion, but not widely practiced in surgery (9). Therefore, surgeons often have to rely on either visual evaluation alone, simply by looking at the colour changes – dark yellow to brown – *in situ* PGs (ISPGs), or the “knife” test to estimate parathyroid perfusion and tissue viability (10,11). However, it may not be objective to distinguish the ischaemic or vital tissues solely depending on their visual examination.

Indocyanine green (IG) is an inert, water soluble, nonradioactive and nontoxic contrast agent that has been used to enhance fluorescence imaging. It enables real-time assessment and direct imaging of tissue perfusion and vascularisation. After intravenous injection, IG is distributed through the intravascular space and rapidly becomes bound to plasma proteins. When illuminated at 806 nm with a low-energy laser, these plasma-bound IG molecules become fluorescent, recorded by a charge-coupled device camera. Since the fluorescence intensity in a focused area is directly proportional to the perfusion of that area, the value of ISPGs measured on the IG fluorescence angiography (IGFA) may provide valuable information about the blood perfusion and the extent of viability of PGs (12,13). Currently, this technique is discussed as a new tool to assess parathyroid perfusion during thyroidectomy (8). In the present study, we aimed to compare the quantitative evaluation of ISPG perfusion by IGFA using a SPY imaging system (Novadaq, Ontario, Canada) or by visual examination performed by surgeon after a total thyroidectomy.

## SUBJECTS AND METHODS

### Patients

This prospective interventional pilot study enrolled forty-three patients aged between 18 and 75 years old, who underwent a total thyroidectomy in our Department of Surgery due to benign or malign symptoms. The duration of the study period includes a total of six months of surgeries. The exclusion criteria were a history of previous operation on the thyroid or neck; symptoms of hypo/hypercalcaemia or hypo/hyperparathyroidism; malignancy which was planned to be dissected on the neck simultaneously; a known iodide allergy and chronic kidney failure. The study was approved by the Bakırköy Sadi Konuk Clinical Research Ethics Board at the University of Health Science in Istanbul, Turkey (No. 20170809). Written informed consent was obtained from all study participants.

### Visual examination

One experienced surgeon performed all the procedures and the surgeon attempted to identify and localise PGs during a total thyroidectomy. The blood perfusion of each PG was scored by the same surgeon through a visual examination scored as 0 (ischaemic tissue), 1 (changed colour) and 2 (normal appearance) before the closure of the wound, according to the literature (14). The surgeon left as many vascularised PGs as possible by cautiously preserving their attached vascular pedicle. Those PGs that could whose blood pedicle could not be preserved or were inadvertently removed during the operation or given a score of 0 by the surgeon were auto-transplanted. For analysis, all patients with at least one scored PG were eligible for analysis.

### Operative techniques

Operative techniques and postoperative care were standardised according to the literature (8,11,15). All thyroidectomies were performed under general anaesthesia by an experienced surgeon. To preserve the blood supply of each PG, a careful dissection was performed. Each terminal branch of the thyroid artery or vein was ligated individually close to the capsule of the distal PG by using 4/0 ties. No energy devices (including electrocautery) were used during dissecting in close proximity to PGs. After the entire thyroid gland was removed, the specimen was examined for any missing PGs. Any PGs inadvertently devascularised or removed or given 0 by the surgeon for blood perfusion were immediately taken out. Excised PGs were immersed in physiologic saline until the final stage of the operation, and then sliced into pieces of 1x1 mm in size. Separated pockets were prepared within sternocleidomastoid muscle fibers on the same side of each 3-4 pieces. After PG slices were implanted in the pocket, the muscle was closed with a 4/0 prolene suture, marked and autotransplanted into the ipsilateral sternocleidomastoid muscle (3).

### Intraoperative IGFA

**Figure 1:**
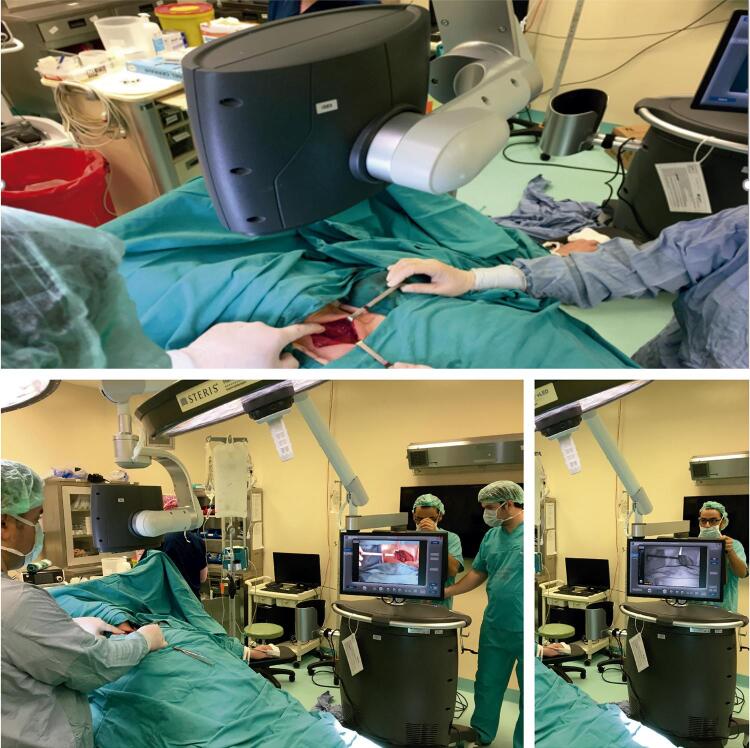
Spy image system in surgery room.

**Figure 2:**
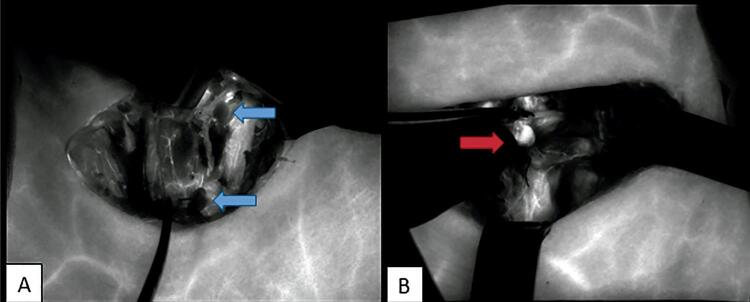
A SPY image of A: the ischemic left parathyroids (blue arrow) B: in situ left parathyroid (red arrow) well-differentiated with indocyanine green fluorescence agent.

After the entire thyroid gland was removed, an intraoperative IGFA was performed using the SPY Fluorescent Imaging system (Novadaq Technologies, Inc, Mississauga, Ontario, Canada). This system consists of an imaging head equipped with a charge-coupled device camera, a laser light source, and a distance sensor ( Figure 1 ). The laser operates at a power density of 40 mW/cm ^2^ and the camera head has a proximity limitation of 5 cm from the area of interest (i.e., the thyroid bed) to prevent overexposure. During each IGFA session, the camera head was positioned approximately 15–20 cm away from the thyroid bed. Once the imaging head was positioned correctly, 3 mL of IG (2.5 mg) was given intravenously by an anaesthetist. Then, the vascular access was washed with 10 ml physiological serum. Real-time fluorescent images of the entire thyroid bed were then recorded at approximately 3 minutes after the injection. Images were recorded by the charge-coupled device camera. For each of PGs, the values of the plateau phase, reached at the maximum radiant intensity, were accepted to represent the perfusion levels and recorded. To confirm whether the structure observed on the real-time images was an ISPG but not a blood vessel or remnant thyroid tissue, the surgeon manually picked up each identifiable ISPG during recording by the device. Since each identifiable ISPG was clipped after the biopsy, the attached metal clips/ties were coupled with the identification of *in situ* PG on the images. After all identifiable ISPGs were clearly seen, the recording was stopped. The SPY system accepts the moment when no fluorescence is visualised as 0, and scores any fluorescence according to the radiant intensity (perfusion) ( Figure 2 ). The highest three numerical scores of each PG for each patient were averaged at the plateau phase of the recorded video and reported as the blood perfusion score (PS) of that PG. This scoring was finalised while the patient was still under anaesthesia. All PGs were evaluated and scored separately.

### Postoperative management and follow-up

Serum parathyroid hormone (PTH) and Calcium (Ca) levels were measured preoperatively for three times, immediately after operation (within 24 hours), and on the following three months. Hypocalcaemia was defined as serum adjusted Ca < 8.5 mg/dL, measured within 24 hours of total thyroidectomy (range: 8.5–12.5 mg/dL). Oral Ca ± calcitriol was not routinely given to the patients unless the level of adjusted Ca was under 8.5 mg/dL or the patients had postoperative symptoms. Under these circumstances, two ampules of intravenous Ca (Calcium Picken 10% ampule containing 225 mg Calcium Gluconate Monohydrate) were injected twice within the first 24 hours. If the symptoms were not relieved, the injection of Ca was resumed. If needed, oral Ca (Calcimax-D3 ^®^ containing 2500 mg calcium carbonate and 880 IU vitamin D3; Basel Ilac, Istanbul, Turkey) and/or oral calcitriol (Rocaltrol ^®^ containing 0.25 µg calcitriol; Deva Ilac, Istanbul, Turkey) were prescribed twice daily until the first clinic visit (15). Patients requiring Ca ± calcitriol supplements after their first postoperative visit were followed-up, and their Ca and PTH levels were checked every 4 weeks until they could maintain normocalcaemia and normoparathyroid without supplements (8,15).

### Laboratory methods

Serum albumin-adjusted Ca and phosphate levels were measured in the laboratory by standard methods using the Roche Diagnostics Modular Analytic system (Roche Diagnostics, Indianapolis, IN). PTH level was measured by an Access 2 immunoassay system (Beckman Coulter, Brea, CA), and the inter- and intra-assay CVs were 5.8% and 4.5%, respectively. The normal range for serum PTH level was accepted to be between 12-88 pg/mL.

### Statistical analysis

The Fisher exact test was used to compare the categorical variables, while the Mann-Whitney U test was used to compare the continuous variables. For correlation between two continuous variables, the Pearson correlation test was performed. All statistical analyses were performed using GraphPad InStat Version 3.6. P < 0.001 was considered statistically significant.

To calculate the cut-off value for PSs of different locations of PGs, the Number Cruncher Statistical System 2007 (NCSS, Kaysville, Utah, USA) program was used. Diagnostic screening tests (sensitivity, specificity, PPV, NPV) and receiver operating characteristic (ROC) curve analysis were used to determine the cut-off for the parameters. Again, P < 0.001 was considered statistically significant.

## RESULTS

All of the 43 consecutive patients underwent a successful IGFA after a primary total thyroidectomy. Table 1 shows the baseline characteristics of the thyroidectomy patients. The operative indications of these patients were benign pathology (76.7%) with a multinodular goitre (44.2%) and hyperthyroidism (32.6%), and malignancy (23.3%). Among these patients, 129 PGs were identified and scored by the same surgeon. Moreover, only one patient (2.3%) had one identifiable PG, six (13.9%) had two PGs, 28 (65.2%) had three PGs and eight (18.6%) patients had four PGs, confirmed by both the surgeon and SPY machine, which were eligible for analysis. None of these patients had any parathyroid tissue or gland inadvertently found in the excised thyroid specimen. PG auto-transplantation was decided in 16 (37.2%) patients. Two patients had two PGs to be transplanted and the others had one PG transplanted. The locations of excised PGs were left upper, left lower, right upper and right lower with a ratio of 24.8%, 25.6%, 25.6% and 24.0%, respectively.

**Table 1:** Baseline characteristics of the patients undergoing total thyroidectomy

Parameters	N = 43
Age (Mean± SD) [Min-Max]	44.48 ± 12.31 [20-70]
Sex (Male:Female) (%)	8:35 (18.6:81.4)
Operative indication (%)	
Benign pathology Multinodular Goiter Hyperthyroidy Malignancy	33 (76.7) 19 (44.2) 14 (32.6) 10 (23.3)
# of patients with (%)	
1 ISPG 2 ISPGs 3 ISPGs 4 ISPGs	1 (2.3) 6 (13.9) 28 (65.2) 8 (18.6)
# of patients with (%)	
No PG autotransplanted PG autotransplanted	27 (62.8) 16 (37.2)
Location of the visualized PGs (%)	
Left upper Left lower Right upper Right lower	32 (24.8) 33 (25.6) 33 (25.6) 31 (24.0)

PG: parathyroid gland; ISPG: in situ parathyroid gland.

Biochemical parameters of the patients ( Table 2 ) showed that 22 patients (51.2%) developed hypocalcaemia within 24 hours postoperatively, and all patients were able to recover and achieve normocalcaemia within 3 months of surgery. All patients were weaned off Ca ± calcitriol supplements within 4 weeks of operation. Five patients (11.6%) developed a temporary hypoparathyroidism within the first 24 hours postoperatively, but subsequently achieved normal parathyroid activity within 3 months.

**Table 2:** Biochemical parameters of the patients undergoing total thyroidectomy

Parameters	Preoperative	Postoperative (at first day)	P value
Adjusted	8.99 ± 0.48	8.43 ± 0.63	**< 0.0001**
Ca (mg/dL)	[7.92-10.2]	[7.26-9.8]	
Mean ± SD [Min-Max]			
# of patients with	5 (11.6%)	22 (51.2%)	**0.0001**
hypocalcemia			95% CI
(< 8.5 mg/dL)			[1.54-7.49]
PTH (pg/mL)	48.35 ± 13.83	35.11 ± 19.16	**< 0.0001**
Mean ± SD	[20.0-72.0]	[5.0-79.0]	
[Min-Max]			
# of patients with hypoparathyroidy (< 12 pg/mL)	0	5 (11.6%)	0.055
hyperparathyroidy (> 88 pg/mL)	0	0	1.000

Ca: calcium; PTH: parathormone; CI: confidence interval.

Table 3 provides the PSs for each identified PG according to the location and glandular appearance. The mean IGFA-PS of the left upper six PGs (18.74%), given visual score of 0 by the surgeon, was 44.83 ± 5.46 [40-54], and this significantly correlated with the visual PS score (P < 0.001). The mean IGFA-PS score of 11 PGs (34.38%) in the left upper location, scored as 1 by the surgeon, was 96.55 ± 19.06 [66-116], and for the 15 PGs (46.88%) scored as 2 the IGFA-PS was 156.53 ± 49.21 [70-253]. These increased IGFA-PSs in the left upper location were also significantly correlated with visual PSs (P < 0.001).

**Table 3:** Correlation of the blood perfusion scores of the patients undergoing total thyroidectomy

Location of PG	Surgeon’s PS		SPY-PS	P value

	Score	N (%)	Mean ± SD [MIN-MAX]	
Left upper	0 1 2	6 (18.74) 11 (34.38) 15 (46.88)	44.83 ± 5.46 [40-54] 96.55 ± 19.06 [66-116] 156.53 ± 49.21 [70-253]	**< 0.0001** 95% CI [0.59-0.89]
Left lower	0 1 2	3 (9.09) 14 (42.42) 16 (48.48)	55.0 ± 13.23 [45-70] 95.29 ± 37.30 [36-144] 159.19 ± 45.03 [90-242]	**< 0.0001** 95% CI [0.50-0.85]
Right upper	0 1 2	3 (9.09) 14 (42.42) 16 (48.48)	46.33 ± 14.84 [30-59] 77.0 ± 16.56 [50-106] 148.88 ± 32.86 [116-224]	**< 0.0001** 95% CI [0.68-0.91]
Right lower	0 1 2	6 (19.35) 16 (51.61) 9 (29.03)	48.17 ± 10.03 [35-62] 89.75 ± 23.20 [48-138] 170.44 ± 29.33 [128-218]	**< 0.0001** 95% CI [0.75-0.94]
Total	0 1 2	18 (13.95) 55 (42.64) 56 (43.41)	48.58 ± 4.49 [30-70] 89.65 ± 8.93 [36-144] 158.76 ± 8.93 [70-253]	**< 0.0001** 95% CI [0.72-0.85]

CI: confidence interval; PG: parathyroid gland; PS: perfusion score.

Regarding the left lower location ( Table 3 ), the mean IGFA-PS of three PGs (9.09%), scored as 0 by the surgeon, was 55.0 ± 13.23 [45-70], and there was a significant correlation with visual PS (P < 0.001). The mean IGFA-PS of 14 PGs (42.42%), scored as 1 by the surgeon, was 95.29 ± 37.30 [36-144], and for the 16 PGs (48.48%) scored as 2, IGFA-PA was 159.19 ± 45.03 [90-242]. Again, these IGFA-PSs in the left lower location strongly correlated with the visual PSs of relevant PGs (P < 0.001).

For the right upper location ( Table 3 ), the mean IGFA-PS of three PGs (9.09%), scored as 0 by the surgeon, was 46.33 ± 14.84 [30-59], and there was a significant correlation with visual PS (P < 0.001). The mean IGFA-PS score of 14 PGs (42.42%), scored as 1 by the surgeon, was 77.0 ± 16.56 [50-106], and for the 16 PGs (48.48%) scored as 2, IGFA-PS was 148.88 ± 32.86 [116-224]. These increased IGFA-PSs in the right upper location also highly correlated with the visual PSs of relevant PGs (P < 0.001).

Considering the right lower location ( Table 3 ), the mean IGFA-PS of six PGs (19.35%), scored as 0 by surgeon, was 48.58 ± 4.49 [30-70], and there was a significant correlation with visual PS (P < 0.001). The mean IGFA-PS score of 16 PGs (51.61%), scored as 1 by the surgeon, was 89.65 ± 8.93 [36-144] and for the nine PGs (29.03%) scored as 2 IGFA-PS was 158.76 ± 8.93 [70-253]. These increased IGFA-PSs in the right lower location were again considerably correlated with the visual PSs of the relevant PGs (P < 0.001).

Across all locations, the mean IGFA-PS of a total number of 18 PGs (13.95%), given a visual PS of 0 by the surgeon, was 48.58 ± 4.49 [30-70]; of a total number of 55 PGs (42.64%) given a visual PS of 1, the mean IGFA-PS was 89.65 ± 8.93 [36-144]; and of a total number of 56 PGs (43.41%) given a visual PS of 2, mean IGFA-PS was 158.76 ± 8.93 [70-253]. These gradually increased IGFA-PSs in all locations were significantly correlated with the visual PSs of relevant PGs (P < 0.001).

In Table 4 , the predictive cut-off values for IGFA-PS were given in four locations of PGs and for total scores of all PGs. According to the results of the ROC analysis, the maximum cut-off value of the patients given a visual PS of 0 was chosen to be 70 (P < 0.0001; 97% CI [0.82-0.99]), and this was interpreted to be a candidate cut-off value for auto-transplantation of PGs. The predictive cut-off value of the total of 129 PGs including all locations was 62 (P < 0.0001; 98% CI [0.93-0.99]).

**Table 4:** ROC analysis of the blood perfusion scores of the patients undergoing total thyroidectomy

Location	AUC	LCI-UCI	p value	Cut-off	Sensitivity	LCI-UCI	Specificity	LCI-UCI	PPV	NPV
Left upper	1.00	0.89-1.00	< 0.0001	> 54	100.00	86.8-100.0	100.0	54.1-100.0	100.0	100.0
Left lower	0.97	0.82- 0.99	< 0.0001	> 70	90.00	73.5-97.9	100.0	29.2-100.0	100.0	50.0
Right upper	0.97	0.85-0.99	< 0.0001	> 59	93.33	77.9-99.2	100.0	29.2-100.0	100.0	60.0
Right lower	0.98	0.85-1.00	< 0.0001	> 62	96.00	79.6-99.9	100.0	54.1	100.0	85.7
Total	0.98	0.93-0.99	< 0.0001	> 62	96.40	91.1-99.0	94.44	72.7-99.9	99.1	81.0

LCI: 95% Lower Confidence Interval; UCI: 95% Upper Confidence Interval.

## DISCUSSION

IG is a safe, reliable and inexpensive dye that has been used for thyroid operations, and also as a useful contrast agent for PG identification (16). IGFA is one of the most recent breakthroughs in the field of intraoperative imaging and has been claimed to modify perioperative surgical management. In fact, it might prevent hypoparathyroidism by limiting trauma to the PGs during surgery, allowing the surgeon to dissect the vasculature of the PG more thoroughly and carefully, eventually leaving a thyroid remnant to preserve the parathyroid tissues (17). Moreover, IGFA allows each gland to be evaluated separately, and does not limit the examination for overall PG function. Another benefit of IGFA is to support decisions to manage the choice of PGs to be auto-transplanted. In this study, 16 out of 43 patients (37.2%) underwent PG auto-transplantation, with a total number of 18 PGs, i.e. two patients had two PGs transplanted and the others had one PG transplanted. In a long-term follow-up study, an analysis of 194 patients having thyroidectomy and simultaneous parathyroid autotransplantation revealed that 104 (54%) patients experienced the symptoms and signs of hypocalcaemia. Parathyroid autotransplantation was successful in 103 (99%) of these 104 cases and resulted in a 1.0% incidence of hypoparathyroidism in this series (18). However, the preservation of vascularity and viability of PGs *in situ* is desirable, hence, an objective scoring system during thyroidectomy may virtually eliminate postoperative hypoparathyroidism.

A drawback of IGFA is the identification of PGs without precise certainty. Even experienced surgeons might misinterpret the glands as other anatomical structures. Thus, some would prefer to give visual scores between 0 and 2, according to the appearance of blood perfusion. In experienced hands, however, the rate of correct identification of a structure as a PG is said to exceed 95% per cent (8). The results of a recent study by Vidal Fortuny and cols. have suggested that a systematic biopsy is not necessary to confirm that the identified structure is a PG (19). They asserted that in less experienced hands, IGFA would be valuable to have a tool to help detect and identify PGs. Thus, in this study, we compared the tissue PSs of IGFA and of visual examination performed by the same surgeon following a total thyroidectomy. Our results showed that the respective IGFA-PS of PGs scored by a surgeon were significantly correlated with the visual PSs of relevant PGs (P < 0.0001). Thus, this study supported the reliability of the IGFA system in total thyroidectomy as a marker of tissue blood perfusion.

As an alternative technique to SPY angiography, one group has recently reported using the PINPOINT camera (Novadaq, Mississauga, ON, Canada) to assess tissue perfusion, but it does not provide a numeric value of perfusion, whereas our system does (14). Another study by Lang and cols. (8) has reported IG as a potential perfusion marker for thyroid operations. They confirmed that the patients with a greatest fluorescence value for their PGs would have greater parathyroid perfusion and residual function and, therefore, would be a lower risk of hypocalcaemia than those with a lower value. They concluded that measuring the average fluorescence intensity did not significantly contribute to the overall predictability relative to the greatest fluorescence used alone. They also suggested that finding one well-perfused PG (with a fluorescence intensity > 150%) during the operation might be sufficient for ruling out the subsequent risk of hypocalcaemia in the future. Thus, in this study, we compared the average PSs measured by the SPY device with visual PSs given by the surgeon during thyroidectomy. We assume that taking the average of three highest fluorescence intensities might be more predictive for the tissue perfusion level than using only visual scores, resulting in better decision-making for auto-transplantation of tissues. Perhaps, future studies could focus on how individual PGs function biochemically when the measured IGFA-PS of a PG is higher than 70.

Another finding worth highlighting is that the predictable cut-off value of tissue perfusion is 70, the maximum IGFA-PS of examined PGs among all locations (P < 0.0001). Ischaemic tissues may be inadvertently confused with PGs during visual examination in a total thyroidectomy, which change their colour even in a well-perfused gland. Our study gave a predictive cut-off value for IGFA-PS as 70 at the maximum edge for patients given a visual PS of 0 (95% CI [0.82-0.99]). So, one can interpret this value as a candidate threshold for auto-transplantation of PGs. Importantly, these tissue PSs may also be used to guide hypocalcaemia and manage hypoparathyroidism. Therefore, measuring the PS during IGFA could be an alternative method for predicting post-operative Ca and PTH levels, also facilitating an early hospital discharge after a total thyroidectomy. Moreover, given its potential in distinguishing between well-perfused and less well-perfused PGs, it allows the surgeon to decide which of the ISPGs should be autotransplanted or left *in situ* .

Given that visual inspection is subjective and unreliable (8), measuring the PS on IGFA could become a new technique for selecting patients for parathyroid auto-transplantation. In other words, those PGs with a IGFA-PS less than 70 could be taken out and autotransplanted while those with IGFA-PS higher than 70 might be better to be left *in situ* regardless of their visual appearance. Lang et al. suggested another potential application of IGFA by performing SPY immediately after completing the first side of a total thyroidectomy. They claimed that this would give the surgeon a better idea of the residual parathyroid function in the first completed side before proceeding to the second side of the operation. We also found this approach to be effective in avoiding a longer-term PTH conditions in the future. However, contrary to our findings, their data did not find a significant correlation between the naked eye (i.e., colour change) and IG assessment. Hence, more advanced studies are needed to define an ISPG as being well-perfused or poorly-perfused based on PS alone. Moreover, it is crucial to understand that IGFA is not a perfect predictive tool for biochemical parameters, as there are contradictory data in the literature. Hereafter, it should also be investigated further by multi-centred prospective studies in the future.

Despite these findings, there were several shortcomings with the present study. First, its applicability in other centres remains unconfirmed, since this was a single institution study conducted by one surgeon. Second, owing to the small number of patients, our pilot study was prone to type II error and it remains unknown how IGFA may affect the rate of PTH in the longer-term. Third, it should be noted that biopsy conduction for the PGs during total thyroidectomy was not in the scope of this study, although it may give a predictive rate of correlation between visual identification and histology. Another negotiable point of this study was the fact that not all patients have the same number of parathyroid glands, and at least one PG is considered to be enough for normocalcaemia and normal parathyroid status. In other words, postoperative laboratory results may not reveal the success of operation. Since the ultimate aim of this study was to score the perfusion of PGs with an objective method, this situation may not be considered to decrease the scientific value of this study. Given that no patients developed any long-term hypocalcaemia or hypoparathyroidism, the IGFA may be a promising operative adjunct in determining residual parathyroid function and predicting the risk of these metabolic events. In planning future applications, this new tool may have a role in selecting patients for parathyroid auto-transplantation and guiding surgeons in their approach to blood perfusion in tissues. Another future perspective would be correlating the scores of IGFA with the change in intra-operative PTH measurements, since reduced PTH levels may be a predictor of post-operative symptomatic hypocalcaemia during thyroidectomy.

Since this clinical trial is a pilot study including preliminary findings of an IGFA scoring system to support decision-making for parathyroid autotransplantation, a sample size estimation or power analysis are not provided, and this lack of data could be considered as limitation. Planning future studies should include a power analysis including larger populations of thyroidectomy patients to confirm our preliminary findings. However, there is no established cut-off value for PG vascularisation in order to determine reimplantation, so this first pilot study is still estimable to give a cut-off value by numeric scoring measured by using an IGFA system. This numeric scoring is suggested to be more objective for the vascularity of glands than visual scoring given by a surgeon.

Another limitation of this study is the lack of a paired analysis of the perfusion scores between baseline and the final value in each group and also in total PGs, to understand the loss of vascularity. However, a paired analysis of perfusion scores in each group is not possible since a baseline value is not applicable to measure before the operation or at the starting point of the operation.

This prospective interventional study should be considered to be a pilot study to suggest that finding one well perfused ISPG with a high PS given by IGFA is a reliable way of predicting the tissue perfusion level and also the need for auto-transplantation of PGs. Total thyroidectomy can be performed safely without routine Ca/PTH measurement, without routine Ca supplementation, and with no risk of long-term hypocalcaemia in patients with at least one well vascularised PG. The next step is to use IGFA not only to predict but also to prevent postoperative hypoparathyroidism.
